# Data Association for Multi-Object Tracking via Deep Neural Networks

**DOI:** 10.3390/s19030559

**Published:** 2019-01-29

**Authors:** Kwangjin Yoon, Du Yong Kim, Young-Chul Yoon, Moongu Jeon

**Affiliations:** 1 School of Electrical Engineering and Computer Science, Gwanju Institute of Science and Technology, Gwangju 61005, Korea; yoon28@gist.ac.kr (K.Y.); zerometal9268@gist.ac.kr (Y.-C.Y.); 2 School of Engineering, RMIT University, Melbourne, VIC 3000, Australia; duyong.kim@rmit.edu.au

**Keywords:** multi-object tracking, data association, deep neural network, long short-term memory network

## Abstract

With recent advances in object detection, the tracking-by-detection method has become mainstream for multi-object tracking in computer vision. The tracking-by-detection scheme necessarily has to resolve a problem of data association between existing tracks and newly received detections at each frame. In this paper, we propose a new deep neural network (DNN) architecture that can solve the data association problem with a variable number of both tracks and detections including false positives. The proposed network consists of two parts: encoder and decoder. The encoder is the fully connected network with several layers that take bounding boxes of both detection and track-history as inputs. The outputs of the encoder are sequentially fed into the decoder which is composed of the bi-directional Long Short-Term Memory (LSTM) networks with a projection layer. The final output of the proposed network is an association matrix that reflects matching scores between tracks and detections. To train the network, we generate training samples using the annotation of Stanford Drone Dataset (SDD). The experiment results show that the proposed network achieves considerably high recall and precision rate as the binary classifier for the assignment tasks. We apply our network to track multiple objects on real-world datasets and evaluate the tracking performance. The performance of our tracker outperforms previous works based on DNN and comparable to other state-of-the-art methods.

## 1. Introduction

Multi-object tracking is of great importance in computer vision for many applications including visual surveillance [1], robotics [2], and biomedical data analysis [3]. Although it has been extensively studied for decades, its practical usage for a real-world environment is still limited. Modern advances in object detection algorithms [4,5,6,7,8] in computer vision make the track-by-detection approach become the mainstream of multi-object tracking (MOT). MOT with track-by-detection necessarily exploits data association between existing tracks and new detections at each frame so that it forms trajectories of multiple objects. Thus, data association results produce sequences of detections with unique identities.

Many algorithms have been developed to solve data association problem in MOT. Several research works reformulated the problem as a graph partitioning problem and solved it using either binary integer programming or minimum cliques optimization [9,10,11]. Another group of recent research works uses network flow-based methods [12,13,14] that solve the problem by finding flows in their network. In addition, many tracking methods exploit the appearance of object to discriminate between objects [15,16,17]. There are also conventional approaches such as joint probabilistic data association (JPDA) [18,19] and multiple hypothesis tracking (MHT) [20,21,22] as well as stochastic filtering approaches [17,23].

In [24], Milan et al. proposed data-driven approximations of the data association problem under recurrent neural network approach using Long Short Term Memory (LSTM) that approximates the marginal distributions of a linear assignment problem. They tested their method with simulated scenarios and showed that their method outperformed the JPDA [25] based methods. However, a limitation of their work is that it can process and produce fixed size of input and output. In contrast, we propose a new method based on a bi-directional LSTM that sequentially processes inputs so that it is able to handle arbitrary-size data association problems. The proposed network is comprised of two parts: encoder and decoder. The encoder is a fully connected network with several layers that learns a feature representation of inputs (the position and size of detection bounding box). The decoder is a bi-directional LSTM that can deal with the input sequence of variable size and help to learn from such data.

**Figure 1:**
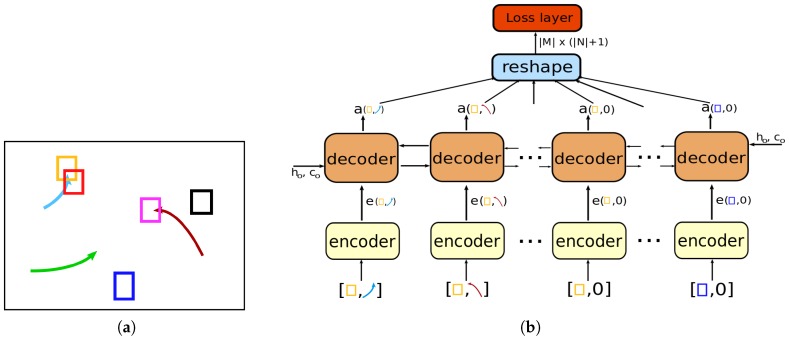
This figure illustrates an example of training sample and model architecture of the proposed deep neural network: (**a**) rectangles are detection bounding boxes in current video frame and each curved arrow represents an existing track; (**b**) each encoder takes bounding boxes of both detection and history of existing track. Then, the decoder reads encoded vectors one by one to generate the association matrix which is fed into the loss layer. The rectangles and arrows in bracket refer to the input pairs in training sample. The same symbols in parentheses after the encoder show their origin (this figure is best viewed in color).

As new detection responses are received at every frame, we have two sets (i.e., a set of detections and a set of existing tracks) to arrange an input to the network. Then, the input of our network is formed by concatenating a detection with an existing track as illustrated in Figure 1b. All possible pairs of detection-to-track at current frame and false alarm for each detection compose a sequence of inputs (a batch of training set). The sequence is consumed by the encoder. Each encoder is a fully connected network with several layers and produces encoded vectors that are sequentially used as inputs to the decoder of our network. The decoder is a bi-directional LSTM with a projection layer solving the association problem for each input in the sequence. Specifically, at the training time, it outputs a sequence of association results by classifying each input into either positive or negative assignment, while at the test time, it outputs a sequence of scores by measuring the quality of the association for each input. The sequence is reshaped to form an association (score) matrix. In Figure 1, an example of the training samples and architecture of the proposed network are described. We show the input pairs (rectangles and arrows) in Figure 1b to clearly specify the data flows. The proposed network is trained using generated samples, by using the ground-truth annotation of a Stanford Drone Dataset (SDD) [1]. We detailed training process in Section 3.2. Finally, the proposed network for data association is used for MOT. The detailed explanation of the MOT algorithm is given in Section 4.2.

Contributions of this paper are as follows: (1) We propose a new deep neural network that can the solve association problem with arbitrary-sized inputs; and (2) we tested the proposed MOT algorithm based on the deigned deep neural network with the real-world datasets, e.g., SDD [1] and MOTChallenge [26]. The proposed network solves data association problems at every frame while it simultaneously produces trajectories. We argue that the result achieves an accuracy comparable to previous works that are similar to ours i.e., data association methods based on deep neural networks which do not exploit the appearance cue. (3) The experiment demonstrates that the proposed network achieves considerably high accuracy as the binary classifier for the assignment tasks.

The remainder of this paper is organized as follows. In Section 2, we review relevant previous works. The detailed explanation of the proposed method is given in Section 3. In Section 4, we state the implementation details and report the experiment results. Finally, we conclude this paper in Section 5.

## 2. Related Works

MOT algorithms are largely classified into two categories: an offline method and online method. In literature, the offline method is getting popular due to superior performance compared to the online method. The offline method takes a sequence of frames as its input. Then, data association for a batch of frames is solved by various optimization algorithms, e.g., network flow [13], shortest path [12,14], linear programming (LP) [27], and conditional random field (CRF) [28]. However, delayed outputs and complexity of the NP-hard (non-deterministic polynomial-time) problem limit its application for real-time requirements.

On the other hand, the conventional approaches based on stochastic filtering such as JPDA [18] and MHT [21] are recently revisited and produce good results due to the good detection quality. Rezatofighi et al. [25] propose an efficient approximation of JPDA to relax the combinatorial complexity in data association. Kim et al. [21] demonstrate that the MHT framework can be extended to include online learned appearance models, resulting in performance gains.

The solution of data association problem described above is obtained by optimizing the objective function. Accordingly, it is required to define an explicit model (e.g., appearance model and motion model) to compute the objective function. Our work is inspired by a series of deep neural network based detection and tracking of multi-objects [24,29,30,31] for the design of objective function. Hosang et al. [29] proposed a learning based non-maximum suppression using a convolutional neural network. The designed network takes bounding boxes of detection responses as input and output exactly one high scoring detection per object. The loss function penalizes double detections for one object during the training procedure. They proposed the GossipNet (Gnet) that jointly processes neighbouring detections so the network has necessary information to report whether an object was detected multiple times. Vinyals et al. [30] propose Pointer Network (Ptr-Net) that provides solutions for three different combinatorial optimisation problems (e.g., convex hull, Delaunay triangulation and the traveling salesman problem). Variable sized inputs are allowed in Ptr-Net. Milan et al. [31] present the end-to-end learning approach for online multi-object tracking using recurrent neural network (RNN). They test their method on real world dataset, MOTChallenge [27], but the performance is inferior to other existing methods. In addition, one drawback of their method is that objects are tracked independently ignoring interactions among objects because they compute the state estimation and data association for one object at a time. The closely related work with ours is [24]. In [24], the solution of combinatorial problems (e.g., marginalisation for data association, feature point matching and the traveling salesman problem) is approximated with an LSTM network. However, their method has one important limitation that it works only on the fixed input and output size.

In practice, the size of data association problem varies with respect to the number of detections and objects that change over time. To handle this issue, we consider a data association problem for a sequence of inputs (a batch) and propose a network to process it sequentially. Hence, our method can learn to solve the data association problem with variable size. Furthermore, we designed our network to consider the context of a sequence when it outputs an association score by using the bi-directional LSTM to exploit future and past information [32].

## 3. Problem Formulation

Let us briefly recap the data association problem of MOT. Data association is a key component of MOT with tracking-by-detection strategy that is used for detection-to-track or detection-to-detection association. Here, we consider association between two sets, i.e., a set of detections $M_{k}$ at time *k* and a set of existing tracks $N_{k-1}$ at time k−1. For notational simplicity, the time index *k* is omitted when no confusion arises. Then, the data association problem is to find correspondences between elements in the two sets representing which detection is generated from which track, while maintaining the one-to-one assignment constraints [33]. The one-to-one constraint is due to the assumption that one object can generate at most one detection. The solution of this problem is described by an association matrix whose elements are logical (binary) variables. The association matrix is usually designed to cope with false positives and false negatives. Thus, the shape of the matrix is (|M|+|N|)×(|M|+|N|). Specifically, the top-left |M|×|N| logical values of the matrix indicate whether the assignment of corresponding detection-to-track pair is made. Similarly, elements of the last |M| columns show the occurrences of false positive for each detection, and the bottom |N| rows are used for indications of the missed detection for each track. Therefore, any solution of the problem is a permutation of the binary matrix satisfying the constraints.

In this paper, the original association matrix is modified by discarding all rows of the missed detections and by collapsing the columns of false positives into a single column (the last column). The size of the original data association matrix can be reduced by introducing an inequality constraint of one-to-one mapping between the existing track and the detection and removing redundant entries of lower part of the matrix. Thus, each row of this last column be $\delta_{m}$ which describes whether the *m*-th detection in *M* is a false positive. Then, the modified association matrix, *Z*, is shaped |M|×(|N|+1). Consequently, the solution of modified association matrix always satisfy following modified assignment constraints:

(1) $$\begin{array}{cc} \sum_{n\in N\cup \delta_{m}}z_{m,n} & =1,\forall m\in M,\\ \sum_{m\in M}z_{m,n} & \leq 1,\forall n\in N,\\ z_{m,n} & \in \{0,1\}, \end{array}$$

where $z_{m,n}\in Z$ is 1 if *m*-th detection and *n*-th track are positive assignment; otherwise, it is set to 0. Note that, in the constraints (1), the false positive of each detection is resolved with the column $\delta_{M}$ (the column for false positives) while missed detection of each track is resolved with inequality. In addition, this smaller matrix helps us use the graphic memory efficiently.

Therefore, the solution of the data association problems is a permutation of the binary matrix satisfying the constraints (1). The solution can be found by computing the maximum assignment set using the score matrix *S* by maximizing the total score of the assignment task. The score matrix *S* has the same shape as *Z* and each element represents an association score of corresponding elements. We show the prediction of our trained network is readily acceptable as the score matrix in Section 4.1 and Section 4.2 since the network is trained to learn the association matrix. Specifically, we maximize the total assignment score over the space of feasible solution of *Z* subject to the constraints (1):

(2) $$\max_{Z}\sum_{m\in M}\sum_{n\in N\cup \delta_{m}}s_{m,n}z_{m,n},$$

where $s_{m,n}\in S$ is a score of the pair *m*-th detection and *n*-th track; in other words, it measures how likely the detection is generated from the track. Once the score matrix is given, the optimization problem of Equation (2) subject to the constraints (1) can be solved in polynomial time, for instance using either the binary integer programming (BIP) or the Hungarian algorithm [24,33].

### 3.1. Model

The proposed network consists of two parts: encoder and decoder (Figure 1). The encoder is the fully connected neural network with several layers. An input of each encoder is a set of pairs of bounding boxes (detection and track/false-positives). Let $d_{m}$ be the *m*-th detection in *M* and $x_{n}$ be the *n*-th existing track in *N*. $d_{m}$ is a vector containing the spatial information (bounding boxes) and the detection confidence. $x_{n}$ consists of a number of detections, i.e., the last *K* detections of the object from *K* previous frames. Then, detection $d_{m}$ and track $x_{n}$ are concatenated and reshaped as a vector to form an input to the encoder. In Figure 1b, the notation [·,·] represents the concatenation operator. The possibility of false positives of given detection is also considered by feeding $\left[d_{m},0\right]$ to the encoder. $\left[d_{m},0\right]$ concatenates the detection $d_{m}$ with zero vectors. A batch of the training set is constructed consisting of all possible detection-to-track pairs including the false positives for each detection at the frame. Then, the encoder processes a whole batch one at a time and consequentially generates |M|×(|N|+1) encoded vectors. For now, let us |Z|=|M|×(|N|+1). For consistency, these |Z| encoded vectors are sequentially processed by the decoder in the form of the association (score) matrix from left-top to right-bottom.

**Figure 2:**
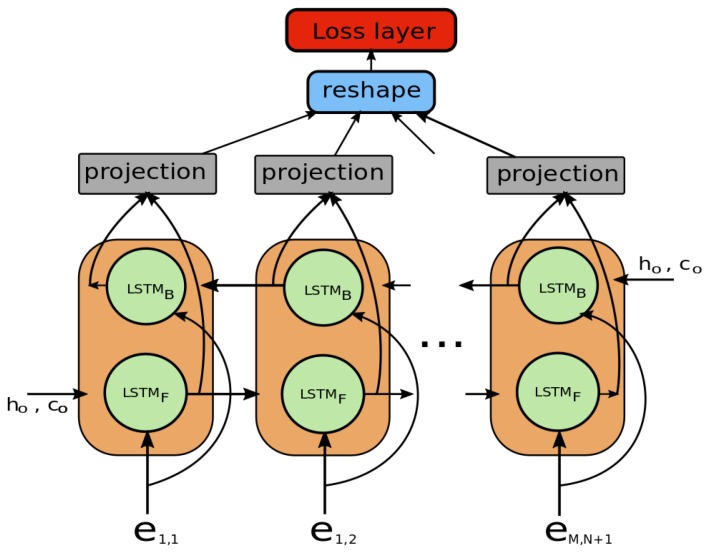
Decoder of the proposed network. Computed hidden states of both forward and backward LSTM are concatenated and inputted to projection layer. Each projection layer outputs an association score of corresponding input.

The decoder of a proposed network consists of a bi-directional LSTM (bi-LSTM) [34,35] and a projection layer on top of the bi-LSTM. The output of decoder is the association (score) matrix. At the training time, the association matrix is used for calculating the loss of the network (Figure 2). The forward LSTM ($LSTM_{F}$) and backward LSTM ($LSTM_{B}$) in the bi-LSTM are implemented as in [35]. The encoded vectors $e_{m,n}$ from the encoder are read by the both LSTMs in two directions, $LSTM_{F}$ for positive direction (left-top to right-bottom of the association matrix) and $LSTM_{B}$ for negative direction (right-bottom to left-top of the matrix) as

(3) $$\begin{array}{cc} {\vec{h}}_{i},{\vec{C}}_{i}= & LSTM_{F}\left(e_{m(i),n(i)},{\vec{h}}_{i-1},{\vec{C}}_{i-1}\right),\\ {\overset{\leftarrow }{h}}_{i},{\overset{\leftarrow }{C}}_{i}= & LSTM_{B}\left(e_{m(i),n(i)},{\overset{\leftarrow }{h}}_{i+1},{\overset{\leftarrow }{C}}_{i+1}\right), \end{array}$$

where m(i) and n(i) are functions that determine the equivalent row number and column number of the *i*-th element in the matrix, respectively, and i=1,2,...,|Z|. For instance, m(j)=1 and n(j)=j, if 0<j≤|N|+1 and j∈N. *h* is the hidden state (after the output gate) and *C* is the cell state. Note that the recursion for $LSTM_{F}$ starts from i=1 while $LSTM_{B}$ starts from i=|Z|. Moreover, we learn the initial hidden state (${\vec{h}}_{0}$ and ${\overset{\leftarrow }{h}}_{|Z|+1}$), and cell state (${\vec{C}}_{0}$ and ${\overset{\leftarrow }{C}}_{|Z|+1}$) using the encoded vectors as

(4) $$\begin{array}{cc} {\vec{h}}_{0}={\overset{\leftarrow }{h}}_{|Z|+1}= & \tanh\left(W_{h}\cdot \bar{e}+b_{h}\right),\\ {\vec{C}}_{0}={\overset{\leftarrow }{C}}_{|Z|+1}= & \tanh\left(W_{c}\cdot \bar{e}+b_{c}\right),\\ \bar{e}= & \frac{1}{\left|Z\right|}\sum_{i=1}^{\left|Z\right|}e_{m(i),n(i)}, \end{array}$$

where $W_{h},b_{h},W_{c}$ and $b_{c}$ are parameters to be trained. The learned initial states are used for both forward and backward LSTMs. We use averaged encoded vector to learn an initial hidden state for LSTMs since it can help to access information of entire inputs at the beginning of decoding procedure. In addition, as introduced in [32,36], the bi-LSTM has an ability to exploit future and past information when it computes the current input. This consideration of information flow in two directions is useful because each association result in the sequence is correlated, i.e., not independent from each other.

Finally, the concatenation of the forward and backward hidden states, $[{\vec{h}}_{i},{\overset{\leftarrow }{h}}_{i}],$ is fed into the projection layer. The projection layer is the fully connected network with several layers where the output of the last layer whose dimension is 1-by-1 is activated by a hyperbolic tangent function. At the training time, the outputs of the projection layers are input into the loss layer as an association matrix, i.e., the solution of given sequence. In Section 4, we specify the architecture of a proposed model with the encoder in Table 1 and the decoder in Table 2, respectively.

### 3.2. Training Proposed Network

**Figure 3:**
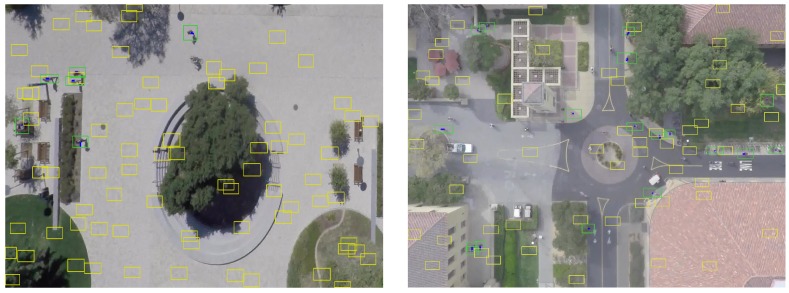
Two examples of training samples. Yellow rectangles are the false positive detections; greens are the true detections. Existing tracks are denoted by blue lines. Note that, since the track length is set to 5 (K=5), they seemed rather short.

To train our network, we have generated training samples from the annotation (ground truth) of the Stanford Drone Dataset (SDD) [1]. The dataset has sixty video sequences in total and each sequence is 6–7 min long in average. We use fifty video sequences to generate training samples and use the remaining sequences for testing purposes. The annotated objects are not just pedestrians, but also bicyclists, skateboarders, cars, buses, and golf carts. Among them, the pedestrian class is used to generate samples. A single training sample is made up of all combinations of detection-to-(track/false positive) assignments at the frame, i.e., a data association problem at the frame. In Figure 3, two examples of training sample at a certain frame are displayed. A detection set *M* for training is formed with a subset of true detections (ground truth) in the frame and plus randomly generated false positive detections. We simulate missed detection by randomly discarding true detections. The discarding probability is determined by $1-P_{D}$, where $P_{D}$ is probability of detection and the number of false positives (alarm) is the Poisson distribution with mean of $\lambda_{FA}$. The existing track set *N* is formed with tracks (objects) from the previous frame. An element in *N* is a track history of an object that consists of the last *K* detections of the object in the past. Some detections in the track history are also discarded according to the discarding probability ($1-P_{D}$). Then, a sequence of assignment problems with the two sets is labeled by a set *L*, where $l_{m,n}\in L$ is 1 if a pair (m,n) is a positive association. Otherwise, it is −1 (negative association), where m∈M and $n\in N\cup \delta_{m}$. For example, if *m*-th detection in *M* is a true false positive, then $l_{m,\delta_{m}}$ is 1, and, consequently, all other $l_{m,}$ are −1 due to the constraints (1).

The loss, L, of our network is weighted mean-squared error (MSE) between labels and predictions of our model. Since every detection is associated only once, the number of negative associations is larger than that of positive associations. To balance the loss, we set weight $w_{m,n}$ to $\frac{|L^{-}|}{|L^{+}|}$ if $l_{m,n}$ is a positive association; otherwise, $w_{m,n}$ is set to one. $|L^{-}|$ and $|L^{+}|$ are the number of negative associations and the number of positive associations in the label set *L*, respectively. In Equation (5), we show the mathematical expression of the loss of our model (L):

(5) $$\begin{array}{c} L=\frac{1}{\left|Z\right|}\sum_{i=1}^{\left|Z\right|}w_{m(i),n(i)}{\left(a_{m(i),n(i)}-l_{m(i),n(i)}\right)}^{2},\\ w_{m(i),n(i)}=\left\{\begin{array}{ccc} \frac{|L^{-}|}{|L^{+}|}, & \mathrm{if} & l_{m(i),n(i)}=1\\ 1, & \mathrm{otherwise} \end{array}\right., \end{array}$$

where $a_{m(i),n(i)}$ is the *i*-th prediction of our model. Therefore, at the training time, our model learns the association matrix, i.e., the solution of given data association problem.

## 4. Results

We demonstrate the efficacy of our network on two experiments. First, we compute the precision and recall to show how accurately our network classifies the positive and negative associations. Second, we apply the data association results from our proposed network for tracking of multiple objects on real-world datasets. The performance of tracking results is reported in both CLEAR-MOT [37] and ID-Measure [38] metrics.

The details of the implementation and the hyperparameters of our network are as follows: to construct training and test samples, a detection $d_{m}$ is defined as a vector (x,y,u,v,w,h,b), where (x,y) is the coordinate of left-top, (u,v) is the coordinate of right-bottom, (w,h) is the size of bounding box and *b* is the detection confidence. The coordinates, width and height are normalized to the range [0,1] with respect to the image dimension. For the false positive detections, *b* is uniformly distributed between (0,1), whereas, for the true detections, *b* is assumed to be normally distributed as $b∼N(b;\mu ,\sigma^{2})$, with mean μ=0.8 and standard deviation σ=0.1. The length of track history *K* is set to 5. For both the training samples and test samples, $P_{D}$ is set to 0.97 and $\lambda_{FA}$ is set to 60.

In Table 1 and Table 2, we show the architecture of the encoder and the decoder, respectively. The encoder consists of three fully connected layers with size 128, 128 and 64 for each layer, respectively. The activation function for first two layers is ReLU (Rectified Linear Unit), while the hyperbolic tangent function is used for the third layer. In Table 1, $|d_{m}|$ is the size of $d_{m}$. We use the bi-directional LSTM of size 128 for the decoder. Then, the projection layer consists of two fully connected layers with size 64 and 1, respectively. The hyperbolic tangent function is applied for activation of the last layer and ReLU is used for the first layer. The learning rate is set to 0.001 and is decreased by 5% every 3000 iterations.

### 4.1. Performance Analysis

**Figure 4:**
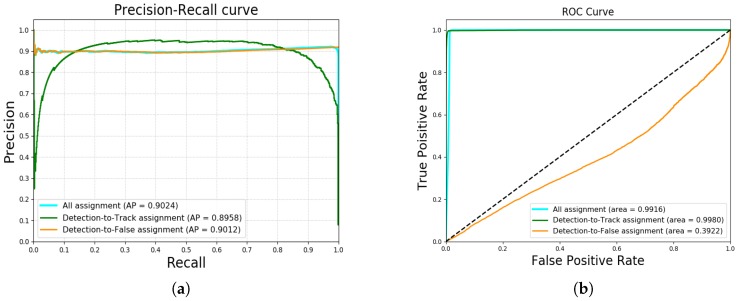
(**a**) precision–recall curve; (**b**) receiver operating characteristic curve.

In this section, we analyze the performance of our network. We regard each prediction in a sequence as the result of binary classification. Specifically, at test time, our network outputs a sequence of association scores of all detection-to-(track/false positive) pairs in a test sample, and we use thresholding these scores at different points to obtain a precision–recall (PR) curve across all test samples. The precision is the fraction of the reported true association that are correct, while the recall is the fraction of the true association that are found. In addition, we also report the receiver operating characteristic (ROC) curve. The PR and ROC curves are illustrated in Figure 4. It is known that the PR curve gives a more informative picture of the performance of the algorithm if there is a large skew in class distribution [39]. In both Figure 4a,b, the yellow curves are drawn from detection-to-false-positive assignments, i.e., $\left(m,\delta_{m}\right)$ while the green curves are generated with detection-to-track assignments, i.e., (m,n),n∈N. The cyan curves are produced from all samples, i.e., $\left(m,n\right),n\in N\cup \delta_{m}$. The distribution of positive associations is very different from each assignment subset. Specifically, the percentage of positive associations in detection-to-false-positive assignments is about 90.9% while the percentage of positive associations in detection-to-track samples is about 1.5%. This is because many false alarms exist in samples due to the high $\lambda_{FA}$ as well as we collapse |M| columns for false positives into a single column ($\delta_{M}$). Accordingly, in Figure 4b, the ROC curve of yellow shows that the predictions of detection-to-false-positive assignments contain fairly many false positives. Nevertheless, ROC curves of cyan and green are close to ideal performance due to its ability of rejecting negative associations. The PR curve shows that the performance of our algorithm achieve a promising result. In Figure 4a, we achieve average precision (AP) of 0.90 in whole test samples. The AP of detection-to-track samples is 0.89, whereas that of detection-to-false-positive samples is 0.90.

### 4.2. Multi-Object Tracking Using a Proposed Network

To demonstrate the benefits of the proposed network, we test the proposed network for data association in multiple objects tracking (MOT). Training of our network is done offline and no further online training is required. Thus, for the MOT task, the loss layer of the network is removed and the output (prediction) of the network is considered to be the score matrix. Furthermore, our tracker does not exploit either the future information or the batch processing, i.e., our tracking system is an online tracker. We use a subset of the matrix (the detection-to-track elements of the score matrix) for data association since many false alarms reside in detection-to-false-positive assignments as we noted in the previous Section 4.1 (Figure 4b). Once the network computes the score matrix, the best assignment in terms of the score is found using the Hungarian algorithm, which satisfies the one-to-one constraint. Note that, in this case, the constrains for detections in Equation (1) is changed to $\sum_{n\in N}z_{m,n}\leq 1,\forall m\in M$. Since the predictions of our network generated by a certain threshold sometimes conflict with the constraints, it is inappropriate to use the output with simple thresholding as the association matrix. A new track is initiated with a detection that is not associated with any existing track. Termination of a track is done if a track misses *D* consecutive frames. We also remove tracks whose lengths are not longer than threshold *T*.

For this task, the performance of trackers is mainly evaluated by the CLEAR-MOT [37]. In addition, we also report the ID-Measure [38]. MOTA (Multiple Object Tracking Accuracy), for which higher accuracy is better, combines three error sources: false positives, missed targets and identity switches, to show the tracker performance. MOTP (Multiple Object Tracking Precision), for which higher is better, is the misalignment between the ground truth and the predicted bounding boxes. MT (Mostly tracked targets), for which higher is better, is the ratio of ground-truth trajectories that are covered by a tracker for at least 80% of their respective life span. ML (Mostly lost targets), for which lower is better, is the ratio of ground-truth trajectories that are covered a tracker for at most 20% of their respective life span. IDsw (identity switch), for which lower is better, is the total number of identity switches. Frag (Fragmentation), for which lower is better, the total number of times a trajectory is fragmented. FP (false positives) and FN (false negatives) are the total number of false positives and negatives (missed targets), respectively. IDP (ID precision), for which higher is better, is a fraction of computed detections that are correctly identified. IDR (ID recall), for which higher is better, is a fraction of ground-truth detection that are correctly identified. IDF1 (ID F-score), for which higher is better, is a ratio of correctly identified detections over the average number of ground-truth and computed detections.

The processing time of our network is dependent on the size of input sequence, which is equal to |M|×(|N|+1). On the SDD dataset, we found that our tracker takes about 500 ms (about two frames per second) when the length of input sequence is 2140, which is the maximum sequence length of the dataset. For the MOTChallenge dataset, the average processing speed is about 172.8 fps (frames per second) while the average sequence length of the dataset is around 42.5. The experiments are performed on the machine with Intel i7-7700K processor, 16 GB RAM and one NVIDIA GTX 1080ti GPU. In addition, our algorithm is implemented with PYTHON and TensorFlow (version 1.4).

#### 4.2.1. Stanford Drone Dataset

**Figure 5:**
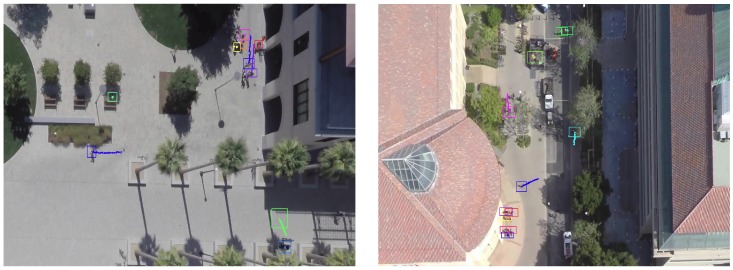
Examples of the qualitative results of multi-object tracking using proposed network on SDD. (**Left**) coupa sequence; (**Right**) gates sequence.

We first track multiple objects on the Stanford drone dataset (SDD). For this experiment, we set parameters as D=3 and T=10, respectively. We also report the performance of a Kalman Filter (KF) tracker as a baseline method. We implement the KF tracker with a linear model [40] algorithm. To track multi-objects with the KF tracker, existing tracks are associated with newly received detection using the Hungarian algorithm after the assignment cost matrix is computed using Euclidean distance between tracks and detections (in this case, the minimum cost will be found). The same track initiation and termination is used as ours. We further report the tracking method of [1], which is a modified version of [41] and solves the MOT problem with a Markov Decision Process (MDP). In [1], they replaced the linear motion model with their social forces model. In addition, we show the result of [2] which jointly reasons on multiple cues (appearance, motion, interaction cues) over a specific period of time using LSTMs. Note that [2] with multiple cues is the method that produces the second best performance on the test set of a MOTChallenge dataset (Section 4.2.2). In this experiment, the performance of tracking results is measured by the CLEAR-MOT [37] and ID-Measure [38]. In Table 3, the reported results are aggregated over all video sequences in the testset. The percentage of Rcll, Prcn, MT and ML are shown in Table 3 to match with the tracking results from [1,2]. Since references [1,2] do not share the detection input, both our method and KF tracker take different input from theirs. The large gap (21.9%) between ours and [1,2] in MOTA might result from the difference of detection inputs. However, the highest MOTA score of our method indicates the excellence of the tracker that uses a proposed data association method. Furthermore, our method produces better results than KF trackers for all metrics except the MOTP because our method constructs trajectories by merely linking detections at each frame without any prior motion. Since a KF tracker corrects the trajectory after the measurement update stage, which results in precise localization, it achieves 2.1% more than ours in MOTP. Our method recovers most of the life span of objects, and consequently it achieves the best performance in terms of mostly tracked objects (MT) in addition to not producing any mostly lost objects (ML). Examples of the qualitative results of our method are shown in Figure 5.

#### 4.2.2. MOTChallenge

We further test our data association network by tracking pedestrians on MOTChallenge dataset [26]. Before testing our network on the dataset, we tried fine-tuning the model using training samples of MOTChallenge dataset. However, we confirm that the fine-tuning does not improve the tracking performance on the validation set (for the fine-tuning task, we isolate the validation set from training set). Accordingly, we keep using the model trained with SDD. We believe that the small size of training samples makes the training procedure difficult.

The MOTChallenge dataset consists of 22 video sequences which are separated into 11 sequences for training and 11 sequences for testing. In all videos, tracking is performed using publicly available detections. Sequences in the dataset are very different from each other, such as view point, target motion, camera motion and person density. These variations make the dataset more challenging. In addition, the annotations of the testing sequences are not released and the evaluation of the testset is performed on a server (https://motchallenge.net/results/2D_MOT_2015/). We use the training set to find the best parameter for the tracking task, i.e., *D* and *T*, by accomplishing higher MOTA as possible. This might lead our tracker to suffer from the precision and FP on the training set (precision and FP are related to each other). On the other hand, however, our tracker makes good scores in the recall and FN as it achieves high MOTA at the same time. This shows the strong point of our method that our network tends to associate true detections more frequently than false positive detections. It is underpinned by the outstanding classification performance in Section 4.1. We set *D* and *T* to 1 and 10, respectively. In Table 4, Table 5 and Table 6, performance of our tracker is generated with D=1 and T=10.

In Table 4, we compare the tracking results on the training set with other baseline methods [25,31] which are related to us. As noted in [25,31], the methods use the same strategy of track initiation/termination, which is similar to our approach mentioned in Section 4.2.1. Note that, in Table 4, all methods including ours only use the spatial information, e.g., detection bounding boxes, but not exploit any other information, e.g., the appearance cue. Both RNN_HA and RNN_LSTM are the online trackers with the same learned motion model using RNN (recurrent neural network), but the difference between them is the data association method [31]. Ref. [25] is the offline tracker unlike [31] and ours. In Table 4, our tracker achieves the best score in terms of MOTA, which is the main criterion of the MOTChallenge.

**Figure 6:**
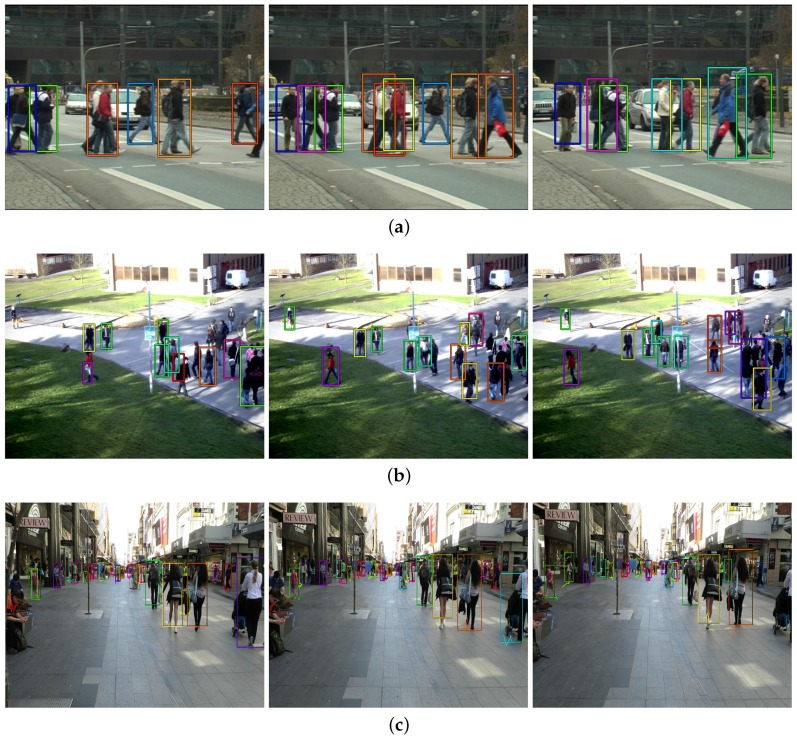
Some examples of the qualitative results of multi-object tracking using the proposed network on the test set of MOTChallenge dataset. From the left to the right, (**a**) TUD-Crossing sequence: the results are sampled at frame 30, 42 and 54, respectively; (**b**) PETS09-S2L2 sequence: the results are sampled at frame 352, 360 and 368, respectively; (**c**) ADL-Rundle-1 sequence: the results are sampled at frame 221, 231 and 241, respectively. The color of each bounding box indicates the person identity.

We show the tracking results on the test set in Table 5 (the date of submission: 21 September 2018). All results in Table 5 are generated using the public detection set as an input. The first two rows are the top two competitors [2,42] on the leader board sorted by MOTA that exploit not only the spatio-temporal cues but also the appearance (visual) cues. We gather trackers (including ours) that are not using any appearance cue on the following rows for fair comparison. The margins in MOTA score between first two rows and the others might results from the existence/absence of the visual information. The visual information of the MOTChallenge dataset is an important information since all targets of the dataset are pedestrians. Human also tracks people by discriminating person-of-interest from others using their appearance. However, since there are many applications that can not utilize such features, e.g., cell or animal tracking, making a good tracker only with spatio-temporal cues is also an importance line of research. In Table 5, Our network performs favourably compared to the other methods using spatio-temporal cues ([31,43,44,45]) and even comparable to [25] which is the offline tracker. Our tracker demonstrates the best processing speed by achieving 172.8 fps. In addition, even if our tracker suffers from the FP on the training set (Table 4), it overcomes the limitation on the test set (Table 5). Specifically, the FP of our tracker is even lower than that of RNN_LSTM in Table 5. This confirms that our tracker is good at rejecting unlikely detection-to-track assignment as we mentioned in Section 4.1. We also add Figure 6 to show qualitative results of our proposed network for MOTChallenge dataset.

Furthermore, to clearly show the power of our method, we compare ours with AMIR15(M + I) and AMIR15(M). The full implementation of [2], i.e., AMIR15, uses multiple cues, namely the appearance (A), motion (M), and interactions (I), while AMIR15(M + I) is limited to use motion and interactions. Likewise, AMIR15(M) is limited to use only the motion cue. We conduct this experiment on a subset of the training set to match with the experiment in [2]. We follow the same rule as in [2] to compose the subset. In Table 6, the results show that the performance of our method is even better than that of AMIR15(M + I) if [2] do not use the appearance information. Note that the full implementation of [2] (AMIR15) holds the second rank among all trackers in Table 5. The results clearly demonstrate that our tracker that uses the spatial information (the position and size of detection bounding boxes) and the detection confidence is outstanding.

## 5. Conclusions

We have proposed a new deep neural network architecture that is able to solve the data association problem of MOT. The proposed network consists of an encoder and decoder. The encoder takes the spatial information and the detection confidence of both a detection and an existing track as an input. It is a fully connected network with several layers while the decoder is a bidirectional LSTM with the projection layer that outputs the association (score) matrix of a given input sequence. At the training time, our model learns the association matrix, i.e., the solution of a given data association problem. Once the training network is finished, the prediction of our network is considered the score matrix. With the score matrix, the maximum assignment set with the maximal total score is found to solve the data association problem for MOT. The experiments show that our proposed network achieves outstanding results on assignment tasks. Furthermore, we show that it can accurately associate detections across time to form trajectories of multiple objects.

For the future work, we plan to apply the convolutional neural network to the encoder in order to capture the appearance feature of objects. In addition, we also have a plan to train our network with more challenging datasets, such as a recent version of the MOTChallenge dataset [26,46] and DukeMTMC dataset [38]. We will further investigate the online MOT framework, e.g., MHT [20,21,22], in order to combine them with our method.

## Figures and Tables

**Table 1:** The architecture of the encoder.

Layer	Type	Input	Output	Activation
1	fully-connected	$|d_{m}|\times \left(K+1\right)$	128	ReLU
2	fully-connected	128	128	ReLU
3	fully-connected	128	64	tanh

**Table 2:** The architecture of the decoder.

Layer	Type	Input	Output	Activation
4, 5	bi-LSTM	64	128	−
6	fully-connected	128	64	ReLU
7	fully-connected	64	1	tanh

**Table 3:** The results on the stanford drone dataset (SDD). The first three columns are for the ID-Measure and the remaining columns are for the CLEAR-MOT. The boldface represents the best score.

Tracker	IDF1	IDP	IDR	MOTA	MOTP	Rcll	Prcn	MT	ML
[1]	-	-	-	75.6	78.2	86.1	92.6	60.0	23.2
[2]	-	-	-	82.9	80.3	92.3	95.3	85.0	15.2
KF	90.0	90.0	90.0	96.4	**90.6**	98.2	98.2	95.5	3.6
Ours	**90.5**	**90.3**	**90.6**	**97.5**	88.5	**99.0**	**98.7**	**98.9**	**0.0**

**Table 4:** The results on the MOTChallenge training dataset.

Tracker	MOTA	MOTP	Rcll	Prcn	MT	ML	FP	FN	IDsw	Frag
JPDA [25]	23.5	69.0	30.6	**81.7**	7.6	69.6	**2728**	27,707	**109**	**380**
RNN_HA [31]	24.0	68.7	37.8	75.2	10.0	53.4	4984	24,832	518	963
RNN_LSTM [31]	22.3	69.0	37.1	73.56	10.0	52.0	5327	25,094	572	983
Ours	**28.8**	**72.3**	**44.2**	75.7	**14.6**	**51.6**	5663	**22,284**	452	802

**Table 5:** The results on the MOTChallenge test dataset.

Tracker	MOTA	MOTP	MT	ML	FP	FN	IDsw	Frag	fps
AP_HWDPL_p [42]	38.5	72.6	8.7	37.4	4005	33,203	586	1263	6.7
AMIR15 [2]	37.6	71.7	15.8	26.8	7933	29,397	1026	2024	1.9
JPDA [25]	**23.8**	68.2	5.0	58.1	**6373**	40,084	1365	1716	32.6
EAMTTpub [43]	22.3	70.8	5.4	52.7	7924	38,982	833	1485	12.2
RNN_LSTM [31]	19.0	71.0	5.5	**45.6**	11,578	**36,706**	1490	2081	165.2
RMOT [44]	18.6	69.6	5.3	53.3	12,473	36,835	**684**	**1282**	7.9
SMOT [45]	18.2	**71.2**	2.8	54.8	8780	40,310	1148	2132	2.7
Ours	22.5	70.9	**6.4**	61.9	7346	39,092	1159	1538	**172.8**

**Table 6:** Comparison between ours and [2] without the appearance component.

Tracker	MOTA	MOTP	MT	ML	FP	FN	IDsw
AMIR15(M) [2]	19.2	73.7	8.5	68.4	3312	15,023	313
AMIR15(M + I) [2]	22.0	**73.8**	9.8	52.1	**2714**	14,954	298
Ours	**24.5**	73.3	**16.2**	**47.8**	3473	**13,177**	**141**
